# The association of intensity and duration of non-pharmacological interventions and implementation of vaccination with COVID-19 infection, death, and excess mortality: Natural experiment in 22 European countries

**DOI:** 10.1016/j.jiph.2022.03.011

**Published:** 2022-05

**Authors:** Feng Zhou, Tao-Jun Hu, Xiao-Yu Zhang, Keng Lai, Jun-Hu Chen, Xiao-Hua Zhou

**Affiliations:** a Department of Biostatistics, School of Public Health, Peking University, Beijing, China; b Beijing International Center for Mathematical Research, Peking University, Beijing, China; c School of Medicine, Macau University of Science and Technology, Macau; d Guangzhou Chest Hospital, Guangzhou, Guangdong, China; e Guangdong Provincial Institute of Biological Products and Materia Medica, Guangzhou, Guangdong, China

**Keywords:** COVID-19, Non-pharmacological interventions, Vaccine, Dynamic intervention, Time series analysis, Excess mortality

## Abstract

**Background:**

Critical questions remain regarding the need for intensity to continue NPIs as the public was vaccinated. We evaluated the association of intensity and duration of non-pharmaceutical interventions (NPIs) and vaccines with COVID-19 infection, death, and excess mortality in Europe.

**Methods:**

Data comes from Our Word in Data. We included 22 European countries from January 20, 2020, to May 30, 2021. The time-varying constrained distribution lag model was used in each country to estimate the impact of different intensities and duration of NPIs on COVID-19 control, considering vaccination coverage. Country-specific effects were pooled through meta-analysis.

**Results:**

This study found that high-intensity and long-duration of NPIs showed a positive main effect on reducing infection in the absence of vaccines, especially in the intensity above the 80th percentile and lasted for 7 days (RR = 0.93, 95% CI: 0.89–0.98). However, the adverse effect on excess mortality also increased with the duration and intensity. Specifically, it was associated with an increase of 44.16% (RR = 1.44, 95% CI: 1.27–1.64) in the excess mortality under the strict intervention (the intensity above the 80th percentile and lasted for 21 days). As the vaccine rollouts, the inhibition of the strict intervention on cases growth rate was increased (RR dropped from 0.95 to 0.87). Simultaneously, vaccination also alleviated the negative impact of the strict intervention on excess mortality (RR decreased from 1.44 to 1.25).

Besides, maintaining the strict intervention appeared to more reduce the cases, as well as avoids more overall burden of death compared with weak intervention.

**Conclusions:**

Our study highlights the importance of continued high-intensity NPIs in low vaccine coverage. Lifting of NPIs in insufficient vaccination coverage may cause increased infections and death burden. Policymakers should coordinate the intensity and duration of NPIs and allocate medical resources reasonably with widespread vaccination.

## Introduction

As of March 06, 2022, it has been nearly 2 years since WHO declared the COVID-19 outbreak a pandemic [1]. Over 0.44 billion confirmed cases and 5.99 million total deaths have been reported in Europe during this pandemic [2]. Many European healthcare facilities had adopted various measures to prevent and control the pandemic, especially non-pharmaceutical interventions (NPIs) and vaccination.

As a new and highly contagious disease, NPIs are an essential component of epidemic prevention and control in the absence of effective treatment regimens, such as physical distancing and traffic restriction [3], [4], [5], [6], [7]. Most of the previous researches on the effectiveness of specific NPIs came from modeling studies [8], [9], [10], [11] and there was little research based on real-world research [12], [13], [14]. Besides, these existing evidences only focused on the changes in health outcomes before and after interventions implementation, which treated the intervention as a break-point [12], [15]. Obviously, in the context of a prolonged pandemic, due to the insufficient medical resources, the intensity of interventions changes dynamically, and they have cumulative and lagging effects on epidemic prevention and control. Meanwhile, post-introduction vaccines may also vary the effects of NPIs [16], [17], [18], [19]. Therefore, critical questions remain concerning the need for intensity to continue NPIs as the public was vaccinated. It is important to explore the effects of the intensity and duration of NPIs and the interactive impact of the vaccine on epidemic prevention and control, which provides guidance for policymakers in the allocation of medical resources, specifically in resource-scarce periods or regions.

In addition, existing studies often adopted indicators that reflect the status of the epidemic as the outcome, such as effective transmission rate and the number of cases, etc. [12], [13], [14], [20], [21]. However, the other impacts caused by pandemics were ignored, such as excess mortality. Excess mortality is a more comprehensive measure of the total impact of the COVID-19 pandemic on deaths. It refers to the number of deaths from all causes during pandemic above and beyond what we would have expected to see under ‘normal’ conditions, including the deaths from other causes that are attributed to the transfer and lack of medical resources and negative of strong restrictive intervention during the pandemic [22], [23], [24], [25], [26]. Therefore, excess mortality also should be considered to comprehensively identify the impact of the intensity and duration of NPIs and vaccination on the prevention and control of the epidemic.

In this study, we aimed to evaluate the independent and added effects of intensity and duration of interventions on the prevention and control of the pandemic in Europe, including on new cases, new COVID-19 death, and excess mortality. We further assessed the changes of these effects after the implementation of vaccine policies and the independent effects of vaccination.

## Materials and methods

### Data source

**Fig. 1:**
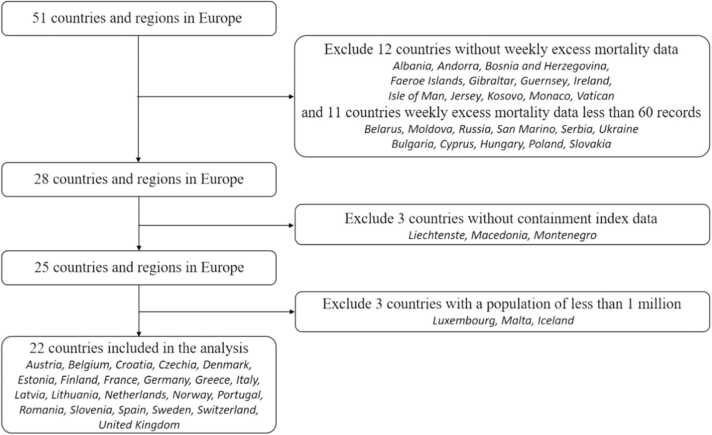
Flowchart of country and region selection.

Data on non-pharmacological interventions and policy responses comes from Oxford Coronavirus Government Response Tracker [27], [28]. We analyzed 22 European countries from January 20, 2020, to May 30, 2021, based on the availability of data, including Austria, Belgium, Croatia, Czechia, Denmark, Estonia, Finland, France, Germany, Greece, Italy, Latvia, Lithuania, Netherlands, Norway, Portugal, Romania, Slovenia, Spain, Sweden, Switzerland, United Kingdom. Specifically, we excluded 12 countries because excess mortality data were not available, and 11 countries since missing data on excess mortality (less than 60 records were available). Similarly, three countries were excluded due to unavailability of Containment and Health Index (CHI). In addition, we exclude 3 countries with a small total population (less than 1 million). The details of sampling countries and flow chart are shown in Fig. 1. The indicators for evaluating the intensity of NPIs using the CHI, a composite measure of twelve of the response metrics (0–100). This index was calculated using these metrics such as school closures; workplace closures; cancellation of public events; restrictions on public gatherings; closures of public transport; stay-at-home requirements; public information campaigns; restrictions on internal movements; and international travel controls; testing policy; the extent of contact tracing; requirements to wear face coverings. In addition, we also focus on the vaccinations against COVID-19, which was obtained from Our World in Data, published by Oxford University [29].

From the COVID-19 Data Repository by the Center for Systems Science and Engineering (CSSE) at Johns Hopkins University, we collected data on the number of daily new confirmed cases and deaths in these countries. Data on excess mortality during the pandemic was sourced from the Human Mortality Database and the UK Office for National Statistics. Other populations and demographic data like population density were from World Bank, and gross domestic product (GDP) per capita was from International Monetary Fund, and hospital beds per thousand were from Eurostat, World Bank, national government records, and other sources [30].

### Definition of available variables

We adopted different definitions of strong interventions (SI) based on intensity criterion (60th, 65th, 70th, 75th, and 80th percentiles) and duration (5, 7, 10, 14, and 21 days) of CHI in each country. Besides, we consider the role of vaccination coverage that people fully vaccinated. According to the current status of people vaccinated, delay in antibody production, and date availability, we separately analyzed the association between the NPIs and the three main outcomes under different vaccine coverage rates (0%, 10%, 20%).

There are three main outcomes of interest in this study. Firstly, we define the cases growth rate by calculating the percentage difference between the current number of daily new cases and the number of daily new cases the day before. Secondly, the death growth rate has also been calculated by the same method. Thirdly, we measure excess mortality using the P-score, which is the percentage difference between the number of deaths in 2020–2021 and the average number of deaths in the same week over the years 2015–2019 [23].

### Statistical analyses

We applied a two-stage time series model to specify the effects of NPIs and vaccination to our main outcomes of interest. To subdivide the effects as for NPIs, we defined the main effect as the independent impact of daily strong interventions and the added effect as the added risk due to the duration of NPIs, which was quantified according to different definitions of strong interventions.

In the first stage regression model, we adopted a linear regression with constrained distributed lag model [31] to estimate main effect, added effect and effect of vaccination policy. For each country, we specify the linear model as,

$P_{j\;}\sim \mathrm{Normal}\left(\mu_{j},\sigma^{2}\right)$

$$\mu_{j}=\alpha_{0}+\mathrm{cb}\left(C_{j},\mathrm{lag}\right)+\beta SI_{j}+\gamma {\text{Vaccination}}_{j}\;,$$

for which $P_{j}$ denotes the value of outcomes of interests on day j. $C_{j}$ is the evaluated CHI on day j, which takes values from 0 to 100; $\mathrm{cb}\left(C_{j},\mathrm{lag}\right)$ is the cross-basis function to fit the accumulative association of non-pharmacological interventions on 0−lag days. $SI_{j}$ is a binary indicator of strong interventions on day j; ${\text{Vaccination}}_{j}$ is another binary variable to represent whether the fully vaccinated people have covered a predetermined percentage of total population up to day j. $\alpha_{0}$ is the country-specific intercept which is time invariant but varies from the countries. β is the coefficient of SI, corresponding to the added effect and γ indicates the effect of vaccination policy. Through a pre-analysis of linear models, we observed heteroskedasticity of which $\sigma^{2}$ may vary with time, specified as $\sigma_{j}^{2}$. We accounted for the heteroskedasticity with a linear regression between $\log\left(\sigma_{j}^{2}\right)$ and the daily accumulative infections of COVID-19.

In the linear regression with constrained distributed lag model above, we left a tuning parameter lag to be determined through a model selection procedure. Selecting the tuning parameter from 1 to 10, we fitted the model respectively. The optimal tuning parameter was decided according to the model performance measured by Akaike information criterion (AIC). The lower estimation of lag suggested that the outcome depended on a shorter period of cumulative interventions.

For the second stage analysis, we pooled all results of 22 European countries using a random-effect meta-analysis. We considered the population density, hospital beds per thousand, and GDP per capita of each country as the moderators to conduct the meta-analysis. All statistical analyses were conducted using R software (version 4.1.0). *P* < 0.05 was considered statistically significant.

## Results

### Descriptive the days of strong interventions

During the pandemic from January 20, 2020, to May 30, 2021, the average days of strong intervention in 22 countries have gradually decreased as the definition of strong interventions becomes stricter (Table 1). Specifically, the average days were more than half of the days (58.98%) under the definition of low threshold (60th percentile) and low duration (5 days). Even with the strict intervention (80th percentile and duration 21 days), the average days that strong interventions lasted accounted for above one-third (35.28%) during the pandemic.

**Table 1:** Mean days and percentage of strong interventions in different definition (intensity and duration) for 22 countries during pandemic.

	Duration	n	Intensity threshold				
			≥ 60th	≥ 65th	≥ 70th	≥ 75th	≥ 80th
Mean days, mean (SD), day	5 days	22	267.95 (40.83)	247.14 (44.48)	218.32 (46.65)	190.27 (47.77)	170.59 (47.28)
	7 days	22	267.45 (41.16)	246.64 (44.82)	217.27 (46.77)	188.95 (48.48)	169.27 (47.43)
	10 days	22	265.73 (41.21)	244.82 (45.5)	217.27 (46.77)	188.55 (48.2)	168.86 (46.96)
	14 days	22	263.68 (41.04)	243.32 (45.01)	215.64 (48.45)	186.91 (49.78)	166.68 (49.04)
	21 days	22	259.91 (43.42)	239.32 (46.75)	209.95 (49.09)	181.45 (51.42)	160.27 (51.90)
Percentage, mean (SD), %	5 days	22	58.98 (9.77)	54.35 (10.03)	47.96 (10.16)	41.86 (10.76)	37.52 (10.51)
	7 days	22	58.87 (9.85)	54.24 (10.11)	47.72 (10.17)	41.57 (10.90)	37.23 (10.53)
	10 days	22	58.50 (9.88)	53.84 (10.27)	47.72 (10.17)	41.49 (10.86)	37.14 (10.45)
	14 days	22	58.05 (9.84)	53.51 (10.17)	47.36 (10.55)	41.12 (11.21)	36.66 (10.92)
	21 days	22	57.22 (10.33)	52.64 (10.56)	46.15 (10.86)	39.96 (11.67)	35.28 (11.60)

#### Impact of NPIs before vaccine policy

**Fig. 2:**
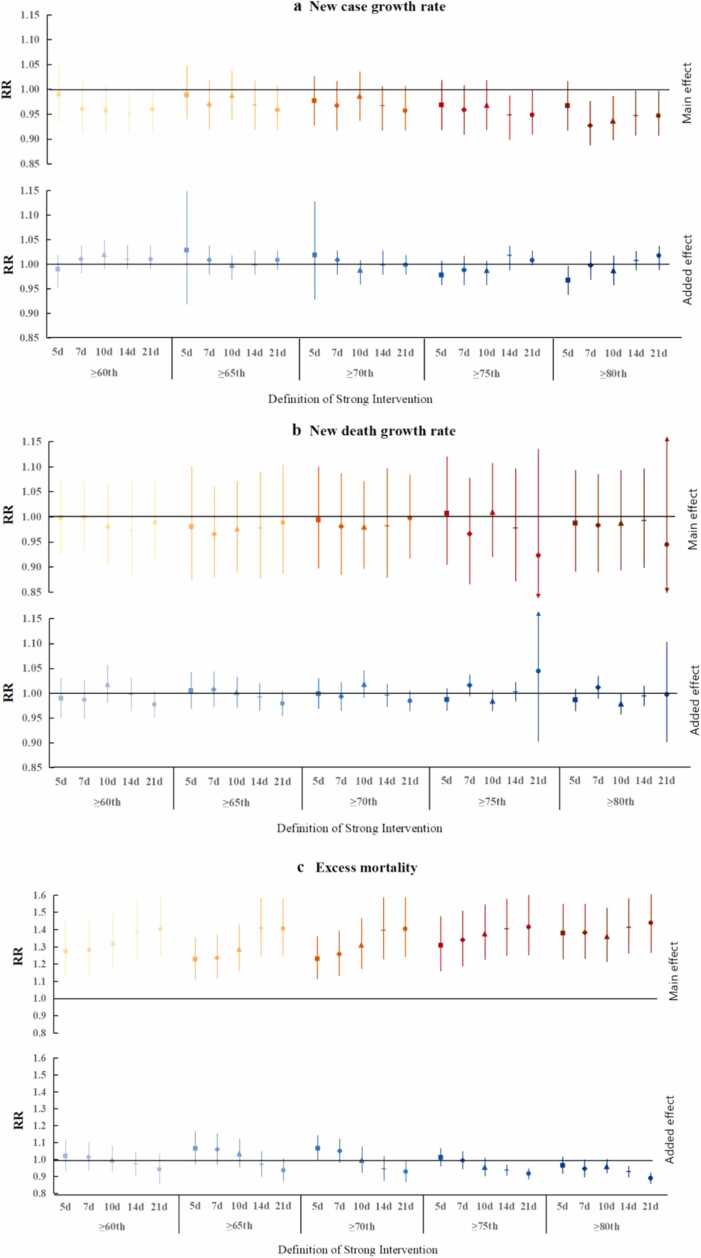
Pooled main and added effects (RR) for three outcomes with random effects model across countries under different strong interventions definitions before vaccine policies impalement. (a) case growth rate, (b) death growth rate, (c) excess mortality.

Fig. 2 presents the pooled main and additional effects of NPIs on case growth rates, death growth rates, and excess mortality under different definitions of strong interventions before the implementation of vaccine policies in 22 European countries. As shown in Fig. 2a, the average main effect of the intervention on the case growth rate was similar and not significant under the 60th, 65th, and 70th of intensity criteria. However, the high-intensity (≥ 80th percentile) was associated with the decrease in the case growth rate, especially 7 days of duration (RR = 0.93, 95% CI: 0.89–0.98) and 21 days of duration (RR = 0.95, 95% CI: 0.91–1.00). In addition, strong interventions had no significant added effect on the case growth rate, regardless of the intensity and duration of intervention.

Although no significant main effect and added effects of the strong interventions were identified on the COVID-19 death growth rate (Fig. 2b). However, the increase in excess mortality was associated with long duration and high intensity of intervention (Fig. 2c). This study found that the strong interventions showed a positive main effect on excess mortality, while there was a negative added effect under some definition. Specifically, the average main effect showed an increasing trend with the duration given the intensity threshold criterion. Such as the RR (risk ratio) raised from 1.28 (95% CI: 1.11–1.46) for lasted 5 days to 1.40 (95% CI: 1.23–1.59) for lasted 21 days at the 60th percentile of intensity criterion. However, these increased trends become small and inconspicuous under high intensity. Furthermore, the intensity also plays an important role in the main effect on excess mortality. As we can see the average main effect also showed an increasing trend as the intensity criterion increases under the same duration. Similarly, these trends were also alleviated as the duration was longer. For example, under the duration of 5 days, the RR raised from 1.28 (95% CI: 1.11–1.46) using 60th percentile to 1.38 (95% CI: 1.23–1.55) using 80th percentile. While under 21 days, the RR raised from 1.40 (95% CI: 1.23–1.59) using 60th percentile to 1.44 (95% CI: 1.27–1.64) using 80th percentile. In contrast, we found the added effect decreased with the duration given the intensity criterion. There was no significant added effect at a lower threshold criterion and shorter duration. When intensity above the 70th (75th, 80th) percentile for at least 14 or 21 days provides a significant negative added effect. Specifically, the RR decreased from 0.96 (95% CI: 0.91–1.02) lasting 5 days to 0.89 (95% CI: 0.85–0.92) lasting 21 days at the 80th percentile of intensity criterion.

### Impact of NPIs and vaccination

**Fig. 3:**
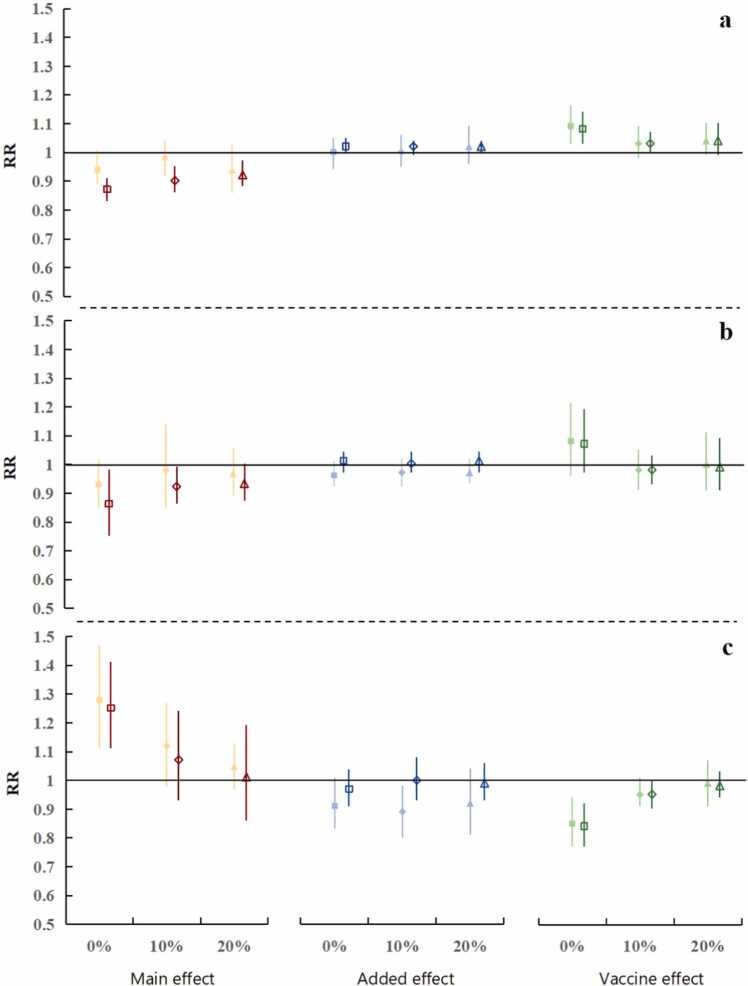
Pooled main, added, and vaccine effects (RR) under the weak (solid) and strict (hollow) intervention and vaccine coverage above 0%, 10%, and 20%. (a) case growth rate, (b) death growth rate, (c) excess mortality.

Table 2 and Fig. 3a summarized the pooled estimates from 22 countries about the impact of NPIs and vaccination on the above three outcomes (case growth rate; death growth rate; and excess mortality). When the vaccine coverage rate was low (0%, 10%, and 20%), the main effect of the strict intervention to control cases was better than weak intervention (RR = 0.94 vs. 0.87; 0.98 vs. 0.90; and 0.94 vs. 0.92, respectively), and the latter was no significant main effect. Moreover, the average main effect of the NPIs for case growth rate was gradually decreased with the increase in vaccine coverage (0%, 10%, 20%), under the strict intervention (RR rose from 0.87 to 0.90 and then to 0.92). While no consistent added effect was found in any scenarios. Similarly, the independent impact of vaccination on case growth rate was not significant, except when the vaccine was just introduced it played an independent role in increasing the case growth rate.

**Table 2:** Pooled main, added, and vaccine effects with tests for heterogeneity (p-value) across countries under the strict and weak intervention and different vaccine coverage (0%, 10%, and 20%) in 22 countries during pandemic.

Vaccination coverage rate (%)	Intensity of intervention	Effect	Case growth rate		Death growth rate		Excess mortality	
			RR (95% CI)	Test for heterogeneity	RR (95% CI:)	Test for heterogeneity	RR (95% CI)	Test for heterogeneity
0	Weak (≥ 60th and 7 days)	Main effect	0.94 (0.89–1.01)	*P* < 0.01	0.93 (0.85–1.02)	*P* = 0.38	1.28 (1.11–1.47)	*P* < 0.01
		Added effect	1.00 (0.94–1.05)	*P* < 0.01	0.96 (0.92–1.01)	*P* < 0.01	0.91 (0.83–1.01)	*P* < 0.01
		Vaccine effect	1.09 (1.03–1.16)	*P* < 0.01	1.08 (0.96–1.21)	*P* < 0.01	0.85 (0.77–0.94)	*P* < 0.01
	Strict (≥ 80th and 21 days)	Main effect	0.87 (0.83–0.91)	*P* = 0.90	0.86 (0.75–0.98)	*P* < 0.01	1.25 (1.11–1.41)	*P* < 0.01
		Added effect	1.02 (1.00–1.05)	*P* = 0.05	1.01 (0.97–1.04)	*P* = 0.18	0.97 (0.91–1.04)	*P* < 0.01
		Vaccine effect	1.08 (1.03–1.14)	*P* < 0.01	1.07 (0.97–1.19)	*P* < 0.01	0.84 (0.77–0.92)	*P* < 0.01
10	Weak (≥ 60th and 7 days)	Main effect	0.98 (0.92–1.04)	*P* = 0.01	0.98 (0.85–1.14)	*P* = 0.38	1.12 (0.98–1.27)	*P* < 0.01
		Added effect	1.00 (0.95–1.06)	*P* < 0.01	0.97 (0.92–1.02)	*P* = 0.12	0.89 (0.80–0.98)	*P* < 0.01
		Vaccine effect	1.03 (0.98–1.09)	P < 0.01	0.98 (0.91–1.05)	*P* < 0.01	0.95 (0.91–1.01)	*P* < 0.01
	Strict (≥ 80th and 21 days)	Main effect	0.90 (0.86–0.95)	*P* = 1.00	0.92 (0.86–0.99)	*P* = 0.22	1.07 (0.93–1.24)	*P* < 0.01
		Added effect	1.02 (0.99–1.04)	*P* = 0.04	1.00 (0.97–1.04)	*P* = 0.08	1.00 (0.93–1.08)	*P* < 0.01
		Vaccine effect	1.03 (1.00–1.07)	*P* < 0.01	0.98 (0.93–1.03)	*P* < 0.01	0.95 (0.90–1.00)	*P* < 0.01
20	Weak (≥ 60th and 7 days)	Main effect	0.94 (0.86–1.03)	*P* < 0.01	0.97 (0.89–1.06)	*P* = 0.49	1.05 (0.97–1.13)	*P* < 0.01
		Added effect	1.02 (0.96–1.09)	*P* < 0.01	0.97 (0.93–1.02)	*P* = 0.13	0.92 (0.81–1.04)	*P* < 0.01
		Vaccine effect	1.04 (0.99–1.10)	*P* < 0.01	1.00 (0.91–1.11)	*P* < 0.01	0.99 (0.91–1.07)	*P* < 0.01
	Strict (≥ 80th and 21 days)	Main effect	0.92 (0.88–0.97)	*P* = 1.00	0.93 (0.87–1.00)	*P* = 0.45	1.01 (0.86–1.19)	*P* < 0.01
		Added effect	1.02 (1.00–1.04)	*P* = 0.17	1.01 (0.97–1.04)	*P* = 0.10	0.99 (0.93–1.06)	*P* < 0.01
		Vaccine effect	1.04 (0.99–1.10)	*P* < 0.01	0.99 (0.91–1.09)	*P* < 0.01	0.98 (0.94–1.03)	*P* < 0.01

As we can see in the middle line of Table 2 and Fig. 3b, the strict intervention had a greater association with COVID-19 death growth rate than the weak intervention, and this association gradually reduced with the increase in vaccine coverage (RR rose from 0.86 to 0.92 and then to 0.93). Moreover, there was no substantial association between vaccination and COVID-19 death growth rate. After the rollout of vaccination, the pooled estimates showed an increase of 25% (RR = 1.25; 95% CI: 1.11–1.41) in the excess mortality associated with the strict intervention. Furthermore, the main effect of the strict (and weak) intervention on excess deaths has dropped sharply with the increase in vaccine coverage, from 1.25 to 1.07 and then to 1.01 (1.28–1.12 and then to 1.05). In particular, we found that combated with NPIs, vaccination played an important role in the decrease of excess mortality. Even under the weak intervention, implementation of vaccine policies can effectively reduce 15% of excess deaths (RR = 0.85, 95% CI: 0.77–0.94).

Fig. S1 and Fig. S2 detail the association between the strict and weak intervention and the three main outcomes after vaccination rollouts in each country during the pandemic, respectively. As we can see in Fig. S1a, the decrease in COVID-19 case growth rate associated with the strict intervention was similar in each country. While the effect of the vaccine was different in these countries. For example, the vaccine effectively reduced the cases growth rate in the United Kingdom and the Netherlands (RR = 0.85 and 0.96), but in Greece and Spain, the opposite was true (RR = 1.22 and 1.50). For COVID-19 deaths, the main effect and added effect of the strict intervention were not statistically significant and there were small differences between countries, and only in a few countries the vaccine has a significant effect (Fig. S1b). For excess mortality, we found that the main effects of the strict intervention on excess mortality are mostly positive in most countries. At the same time, the reduction in excess mortality was related to the implementation of vaccine policies in most countries (Fig. S1c). In addition, details of these effects of the weak intervention after vaccination rollouts in each country see Fig. S2.

## Discussion

Our findings suggest that high-intensity and long-duration of NPIs were associated with a decreased daily new cases growth rate before the implementation of the vaccine policies. However, it is also associated with an increase in excess mortality without affecting the COVID-19 death growth rate. Moreover, our study highlights the importance of continued high-intensity NPIs in low vaccine coverage. After the implementation of the vaccine policies, the inhibition of strict intervention on cases growth rate was increased (RR decreased from 0.95 to 0.87), which may be affected by the interaction of vaccines. Simultaneously, vaccine policies also alleviated the negative impact of strict intervention on excess mortality (RR dropped from 1.44 to 1.25). Furthermore, maintaining strict intervention after vaccination rollouts appeared to more reduce the incidence of new cases, as well as avoids more overall burden of death compared with the weak intervention. In addition, we found that vaccines have independent protection in reducing total deaths even if in the low vaccine coverage rate.

This study not only confirms the previous finding that combined NPIs were beneficial effects on control the infection [4], [5], [6], [12], [32] such as physical distancing [8] and traffic restrictions [33], [34] etc., but also highlights the role of intensity and duration of NPIs in absence of vaccines. A modeling study from 16 worldwide countries speculated that dynamic interventions could provide means to reduce the effective reproduction number and keep ICU demands below the national capacities [35]. Our retrospective study also verified that despite the intervention intensity was only more than the 60th percentile, if it lasted for 21 days, it appeared to achieve a similar effect as above the 80th percentile intensity for 7 days in reducing infections. However, this did not mean that long-term continuous lower-intensity interventions were feasible, because it may result in some potential adverse effects, such as an increase in excess mortality instead of COVID-19 deaths. Unlike previous model-based studies, in which NPIs could reduce the COVID-19 deaths [4], [32], our study from real-world did not find this possibly due to the different periods of analysis. In addition, increased excess mortality could be partly explained by increased deaths from other causes during long-term constraints, such as the inability to seek medical attention in time and weakened healthcare systems [22], [23], [24], [25], [36]. Thus, our study suggests that, it is crucial to comprehensively consider the control infection and death burden in the introduction and implementation of NPIs.

Further, our study also found, the longer duration of low-intensity interventions indirectly implied the health facilities were inactive and incapable in responding to the epidemic, which could result in more excess deaths. Although high-intensity interventions had also independently caused a considerable increase in excess deaths. However, in the long run, the cumulative impact on excess deaths would be reversed, which may be due to the delayed effect of NPIs and this effect diminishes gradually over time [21]. Under this situation, we speculated the contribution of added effects of high-intensity interventions on excess mortality may make up for the main effects. These findings were not mentioned in previous studies and it revealed the significance of long-term continuous high-intensity interventions before the vaccination rollouts from a novel perspective.

Our research confirms that the role of vaccine policies in epidemic prevention and control, which were similar to previous studies conducted in Israel [37] and England [38], and based on modeling [16], [17], [19]. Surprisingly, we found that the strict intervention was better than the weak intervention to control case growth in low vaccine coverage, the latter has no statistical association with case growth rate. Likewise, our study found the vaccine increased the protective effect of the strict intervention on new case-control, illustrating the interaction effects of the NPIs and vaccine strategies. Even B.1.1.7 and other variants may drive the continued increases in the case and could negate intervention and vaccination gains in controlling transmission [39]. Moreover, our analysis also showed that despite vaccination did not decrease COVID-19 deaths, it substantially reduced the excess mortality. Inconsistent with previous research that showed vaccination can protect COVID-19 deaths [18], [19], probably because of the low coverage (only 20%) of vaccines available in our analyzes. Meanwhile, this may be related to the natural reduction in deaths caused by other factors (such as old-age-related diseases) after the first and second waves of the epidemic [22], [40], [41]. In addition, the impact of the same intensity and duration of interventions on excess mortality has been reduced after vaccination rollouts. This may result from that the vaccination makes the allocation of limited medical resources more reasonable and alleviates the lack of medical resources [19], and thus reduced excess deaths. Notably, the excess mortality caused by the weak intervention was greater than strict intervention, and the latter can effectively control cases than the former. This also insinuates lifting or removing of NPIs may increase the death burden in the case of insufficient vaccine coverage. These findings provide additional evidence for policymakers on the lifting of NPIs after the vaccination program.

This study focused on the duration and intensity of NPIs on COVID-19 the prevention and control and we pooled data and summarized experience from included 22 countries in the past nearly 15 months. At the same time, we reported the effects of vaccines from real-world data and gave an inspiration from the perspective of excess mortality. However, there are some limitations. First, the association between NPIs and outcome was not a causal effect in this study, which may be affected by unobserved confounding. We have considered as many observable confounding as possible, such as population size, GDP, and other factors. Second, this study cannot clarify which intervention was more effective. This has been researched in many previous studies [5], [6], [7], [8], [42], and further research is needed to provide answers to this question based on real-world data using other statistical analysis. Third, our research was based on the national-level, therefore we were unable to provide a reasonable explanation regarding the association between NPIs and health outcomes at the individual-level, such as adherence of individual behavior. Finally, this study only analyzed 22 European countries since missing data. This non-random sampling may make the sample less representative. Therefore, this study cannot generalize the whole of Europe.

## Conclusion

In conclusion, through retrospective analysis of the comprehensive effects of national containment measures on population health outcomes during the pandemic, this study confirms the positive role of the high-intensity and long-duration intervention in a decrease of the incidence of COVID-19 in Europe. However, we should still be mindful of the excess mortality during the pandemic, which was ignored by the previous research. Notably, we found that despite there was no significant direct effect on control transmission when the low vaccination coverage rate. However, vaccination may have a positive interaction effect with NPIs on control transmission. Meanwhile, vaccine policies were associated with a large reduction of excess mortality and it could indirectly mitigate the negative impact of NPIs on excess mortality. Even so, we could not relax NPIs in insufficient vaccination coverage, which may cause even greater cases and excess deaths. These findings insinuate with the benefit of high vaccine coverage emerge in the future, and healthcare providers can choose a coordinated and effective intensity and duration of intervention.

## Funding

This work was supported by 10.13039/501100001809National Natural Science Foundation of China [Grant no. 82041023] and The Bill & Melinda Gates Foundation [Grant no. INV-016826].

## CRediT authorship contribution statement

**FZ** and **XHZ** designed this study. **FZ, TJH** and **XYZ** collected, analyzed and interpreted the data and results. **FZ** and **TJH** drafted the manuscript. **FZ, XHZ, KL** and **JHC** provided critical revision of the manuscript for important intellectual content for the manuscript. **TJH** and **FZ** conducted the statistical analysis. **XHZ** supervised this study.

## Patient consent for publication

Not required.

## Ethical Approval Statement

Not required. This observational study was based on population, and no measures have been taken on the population by researchers. All analyzed data comes from public databases and does not involve any individual information.

## Competing interests

The authors declare no competing financial interests.

## Appendix A Supplementary material

Figure S1. Main, added and vaccine effects (RR) for three outcomes in 22 countries under the strict intervention (above 80th percentile and lasts at least 21 days) after the vaccination rollouts during pandemic. (a) case growth rate, (b) death growth rate, (c) excess mortality. Figure S2. Main, added and vaccine effects (RR) for three outcomes in 22 countries under the weak intervention (above 60th percentile and lasts at least 7 days) after the vaccination rollouts during pandemic. (a) case growth rate, (b) death growth rate, (c) excess mortality.

## References

[1] World Health Organization. WHO Director-General's opening remarks at the media briefing on COVID-19 – 11 March 2020. [cited 2020 March 12]. Available from: 〈https://www.who.int/dg/speeches/detail/who-director-general-s-opening-remarks-at-the-media-briefing-on-covid-19---11-march-2020〉.

[2] WHO. WHO Coronavirus (COVID-19) Dashboard. World Health Organization. WHO coronavirus disease (COVID-19) dashboard; 2022. [cited 2022 March 8]. Available from: 〈https://covid19.who.int〉.

[3] Hellewell J., Abbott S., Gimma A., Bosse N.I., Jarvis C.I., Russell T.W. (2020). Feasibility of controlling COVID-19 outbreaks by isolation of cases and contacts. Lancet Glob Health.

[4] Davies N.G., Kucharski A.J., Eggo R.M., Gimma A., Edmunds W.J., Jombart T. (2020). Effects of non-pharmaceutical interventions on COVID-19 cases, deaths, and demand for hospital services in the UK: a modelling study. Lancet Public Health.

[5] Chu D.K., Akl E.A., Duda S., Solo K., Yaacoub S., Schünemann H.J. (2020). Physical distancing, face masks, and eye protection to prevent person-to-person transmission of SARS-CoV-2 and COVID-19: a systematic review and meta-analysis. Lancet.

[6] Regmi K., Lwin C.M. (2020). Impact of non-pharmaceutical interventions for reducing transmission of COVID-19: a systematic review and meta-analysis protocol. BMJ Open.

[7] Flaxman S., Mishra S., Gandy A., Unwin H.J.T., Mellan T.A., Coupland H. (2020). Estimating the effects of non-pharmaceutical interventions on COVID-19 in Europe. Nature.

[8] Kucharski A.J., Klepac P., Conlan A.J.K., Kissler S.M., Tang M.L., Fry H. (2020). Effectiveness of isolation, testing, contact tracing, and physical distancing on reducing transmission of SARS-CoV-2 in different settings: a mathematical modelling study. Lancet Infect Dis.

[9] Peak C.M., Kahn R., Grad Y.H., Childs L.M., Li R., Lipsitch M. (2020). Individual quarantine versus active monitoring of contacts for the mitigation of COVID-19: a modelling study. Lancet Infect Dis.

[10] Badr H.S., Du H., Marshall M., Dong E., Squire M.M., Gardner L.M. (2020). Association between mobility patterns and COVID-19 transmission in the USA: a mathematical modelling study. Lancet Infect Dis.

[11] Aleta A., Martin-Corral D., Pastore Y.P.A., Ajelli M., Litvinova M., Chinazzi M. (2020). Modelling the impact of testing, contact tracing and household quarantine on second waves of COVID-19. Nat Hum Behav.

[12] Islam N., Sharp S.J., Chowell G., Shabnam S., Kawachi I., Lacey B. (2020). Physical distancing interventions and incidence of coronavirus disease 2019: natural experiment in 149 countries. BMJ.

[13] Liu Y., Morgenstern C., Kelly J., Lowe R., Group C.C.-W., Jit M. (2021). The impact of non-pharmaceutical interventions on SARS-CoV-2 transmission across 130 countries and territories. BMC Med.

[14] Wong C.K.H., Wong J.Y.H., Tang E.H.M., Au C.H., Lau K.T.K., Wai A.K.C. (2020). Impact of national containment measures on decelerating the increase in daily new cases of COVID-19 in 54 countries and 4 epicenters of the pandemic: comparative observational study. J Med Internet Res.

[15] Saki M., Ghanbari M.K., Behzadifar M., Imani-Nasab M.H., Behzadifar M., Azari S. (2021). The impact of the social distancing policy on COVID-19 incidence cases and deaths in Iran from february 2020 to january 2021: insights from an interrupted time series analysis. Yale J Biol Med.

[16] Borchering R.K., Viboud C., Howerton E., Smith C.P., Truelove S., Runge M.C. (2021). Modeling of future COVID-19 cases, hospitalizations, and deaths, by vaccination rates and nonpharmaceutical intervention scenarios – United States, April–September 2021. MMWR Morb Mortal Wkly Rep.

[17] Patel M.D., Rosenstrom E., Ivy J.S., Mayorga M.E., Keskinocak P., Boyce R.M. (2021). Association of simulated COVID-19 vaccination and nonpharmaceutical interventions with infections, hospitalizations, and mortality. JAMA Netw Open.

[18] Moore S., Hill E.M., Tildesley M.J., Dyson L., Keeling M.J. (2021). Vaccination and non-pharmaceutical interventions for COVID-19: a mathematical modelling study. Lancet Infect Dis.

[19] Giordano G., Colaneri M., Di Filippo A., Blanchini F., Bolzern P., De Nicolao G. (2021). Modeling vaccination rollouts, SARS-CoV-2 variants and the requirement for non-pharmaceutical interventions in Italy. Nat Med.

[20] Jarvis C.I., Van Zandvoort K., Gimma A., Prem K., group C.C.-w., Klepac P. (2020). Quantifying the impact of physical distance measures on the transmission of COVID-19 in the UK. BMC Med.

[21] Li Y., Campbell H., Kulkarni D., Harpur A., Nundy M., Wang X. (2021). The temporal association of introducing and lifting non-pharmaceutical interventions with the time-varying reproduction number (R) of SARS-CoV-2: a modelling study across 131 countries. Lancet Infect Dis.

[22] Banerjee A., Pasea L., Harris S., Gonzalez-Izquierdo A., Torralbo A., Shallcross L. (2020). Estimating excess 1-year mortality associated with the COVID-19 pandemic according to underlying conditions and age: a population-based cohort study. Lancet.

[23] Our World in Data. Excess mortality during the Coronavirus pandemic (COVID-19). [cited 2021 June 30]. Available from: 〈https://ourworldindata.org/excess-mortality-covid〉.

[24] Vestergaard L.S., Nielsen J., Richter L., Schmid D., Bustos N., Braeye T. (2020). Excess all-cause mortality during the COVID-19 pandemic in Europe – preliminary pooled estimates from the EuroMOMO network, March–April 2020. Eur Surveill.

[25] Islam N., Shkolnikov V.M., Acosta R.J., Klimkin I., Kawachi I., Irizarry R.A. (2021). Excess deaths associated with covid-19 pandemic in 2020: age and sex disaggregated time series analysis in 29 high income countries. BMJ.

[26] Modesti P.A., Wang J., Damasceno A., Agyemang C., Van Bortel L., Persu A. (2020). Indirect implications of COVID-19 prevention strategies on non-communicable diseases: an opinion paper of the European society of hypertension working group on hypertension and cardiovascular risk assessment in subjects living in or emigrating from low resource settings. BMC Med.

[27] Coronavirus Government Response Tracker. Policy responses to the coronavirus pandemic; 2021. [cited 2021 June 20]. Available from: 〈https://www.bsg.ox.ac.uk/research/research-projects/covid-19-government-response-tracker〉.

[28] Our World in Data. Policy responses to the coronavirus pandemic; 2021. [cited 2021 June 20]. Available from: 〈https://ourworldinRdata.org/policy-responses-covid〉.

[29] Our World in Data. Coronavirus (COVID-19) vaccinations; 2021. [cited 2021 June 20]. Available from: 〈https://ourworldindata.org/covid-vaccinations〉.

[30] Our World in Data. Data on COVID-19 (coronavirus) by our world in data; 2021. [cited 2021 June 20]. Available from: 〈https://github.com/owid/covid-19-data/tree/master/public/data〉.

[31] Gasparrini A., Guo Y., Hashizume M., Kinney P.L., Petkova E.P., Lavigne E. (2015). Temporal variation in heat-mortality associations: a multicountry study. Environ Health Perspect.

[32] Chiu W.A., Fischer R., Ndeffo-Mbah M.L. (2020). State-level needs for social distancing and contact tracing to contain COVID-19 in the United States. Nat Hum Behav.

[33] Burns J., Movsisyan A., Stratil J.M., Biallas R.L., Coenen M., Emmert-Fees K.M. (2021). International travel-related control measures to contain the COVID-19 pandemic: a rapid review. Cochrane Database Syst Rev.

[34] Burns J., Movsisyan A., Stratil J.M., Coenen M., Emmert-Fees K.M., Geffert K. (2020). Travel-related control measures to contain the COVID-19 pandemic: a rapid review. Cochrane Database Syst Rev.

[35] Chowdhury R., Heng K., Shawon M.S.R., Goh G., Okonofua D., Ochoa-Rosales C. (2020). Dynamic interventions to control COVID-19 pandemic: a multivariate prediction modelling study comparing 16 worldwide countries. Eur J Epidemiol.

[36] Scortichini M., Schneider Dos Santos R., De’ Donato F., De Sario M., Michelozzi P., Davoli M. (2021). Excess mortality during the COVID-19 outbreak in Italy: a two-stage interrupted time-series analysis. Int J Epidemiol.

[37] Haas E.J., Angulo F.J., McLaughlin J.M., Anis E., Singer S.R., Khan F. (2021). Impact and effectiveness of mRNA BNT162b2 vaccine against SARS-CoV-2 infections and COVID-19 cases, hospitalisations, and deaths following a nationwide vaccination campaign in Israel: an observational study using national surveillance data. Lancet.

[38] Lopez Bernal J., Andrews N., Gower C., Robertson C., Stowe J., Tessier E. (2021). Effectiveness of the Pfizer-BioNTech and Oxford-AstraZeneca vaccines on covid-19 related symptoms, hospital admissions, and mortality in older adults in England: test negative case-control study. BMJ.

[39] Galloway S.E., Paul P., MacCannell D.R., Johansson M.A., Brooks J.T., MacNeil A. (2021). Emergence of SARS-CoV-2 B.1.1.7 Lineage – United States, December 29, 2020–January 12, 2021. MMWR Morb Mortal Wkly Rep.

[40] Norgaard S.K., Vestergaard L.S., Nielsen J., Richter L., Schmid D., Bustos N. (2021). Real-time monitoring shows substantial excess all-cause mortality during second wave of COVID-19 in Europe, October–December 2020. Eur Surveill.

[41] Marcon G., Tettamanti M., Capacci G., Fontanel G., Spanò M., Nobili A. (2020). COVID-19 mortality in Lombardy: the vulnerability of the oldest old and the resilience of male centenarians. Aging.

[42] Mendez-Brito A., Bcheraoui C.E., Pozo-Martin F. (2021). Systematic review of empirical studies comparing the effectiveness of non-pharmaceutical interventions against COVID-19. J Infect.

