# Supplementary material for: The association of intensity and duration of non-pharmacological interventions and implementation of vaccination with COVID-19 infection, death, and excess mortality: Natural experiment in 22 European countries
Source: J Infect Public Health. 2022 May;15(5):499–507. doi: 10.1016/j.jiph.2022.03.011 (PMC8944114; doi:10.1016/j.jiph.2022.03.011)
Supplement: Supplementary file 1 — Figure S1. Main, added and vaccine effects (RR) for three outcomes in 22 countries under the strict intervention (above 80th percentile and lasts at least 21 days) after the vaccination rollouts during pandemic. (a) case growth rate, (b) death growth rate, (c) excess mortality. Figure S2. Main, added and vaccine effects (RR) for three outcomes in 22 countries under the weak intervention (above 60th percentile and lasts at least 7 days) after the vaccination rollouts during pandemic. (a) case growth rate, (b) death growth rate, (c) excess mortality. [file mmc1.pdf]

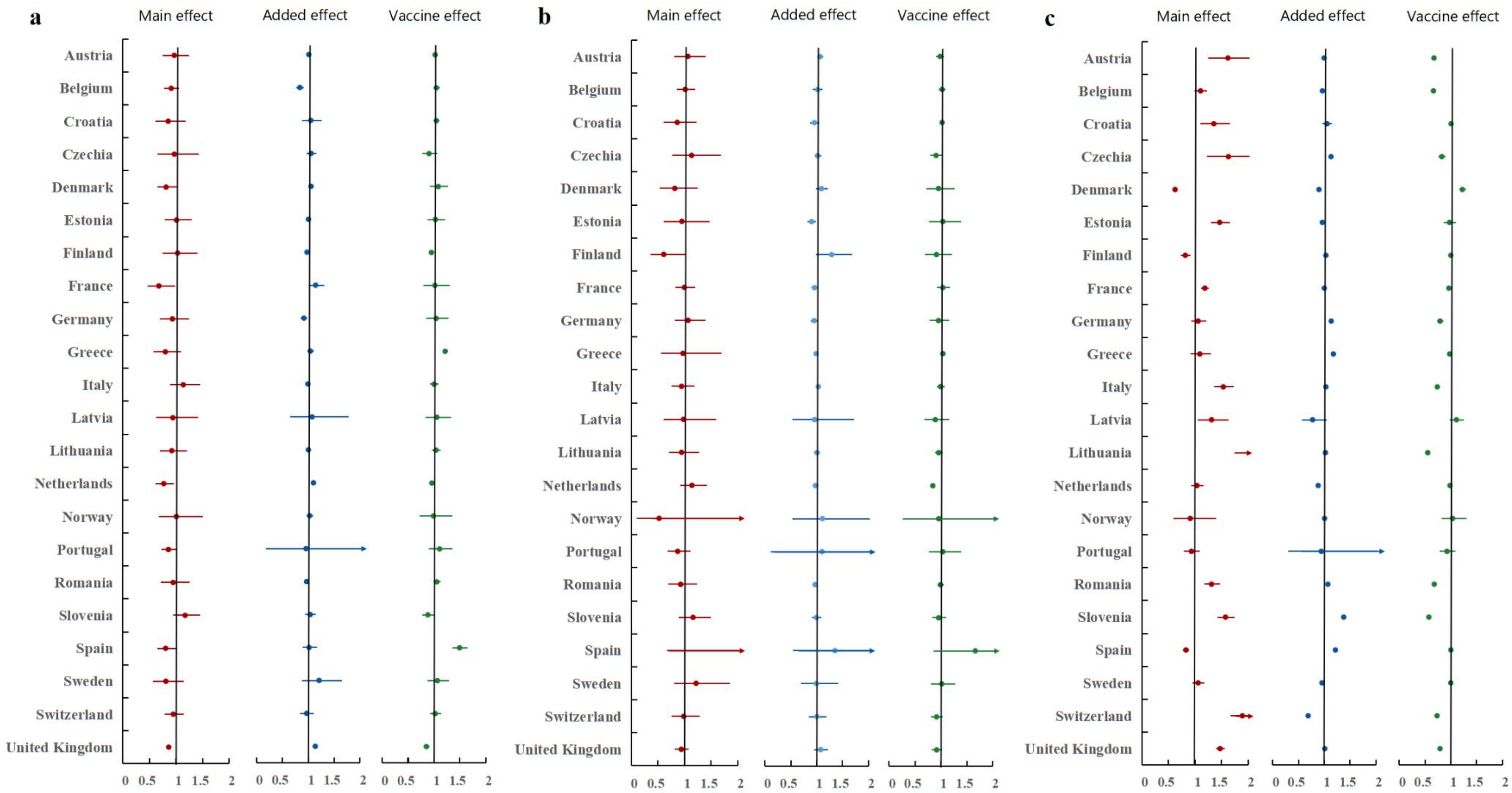

Figure S1. Main, added and vaccine effects for three outcomes in 22 countries under the strict intervention (above 80th percentile and lasts at least 21 days) after the vaccination rollouts during pandemic. (a) case growth rate, (b) death growth rate, (c) excess mortality.

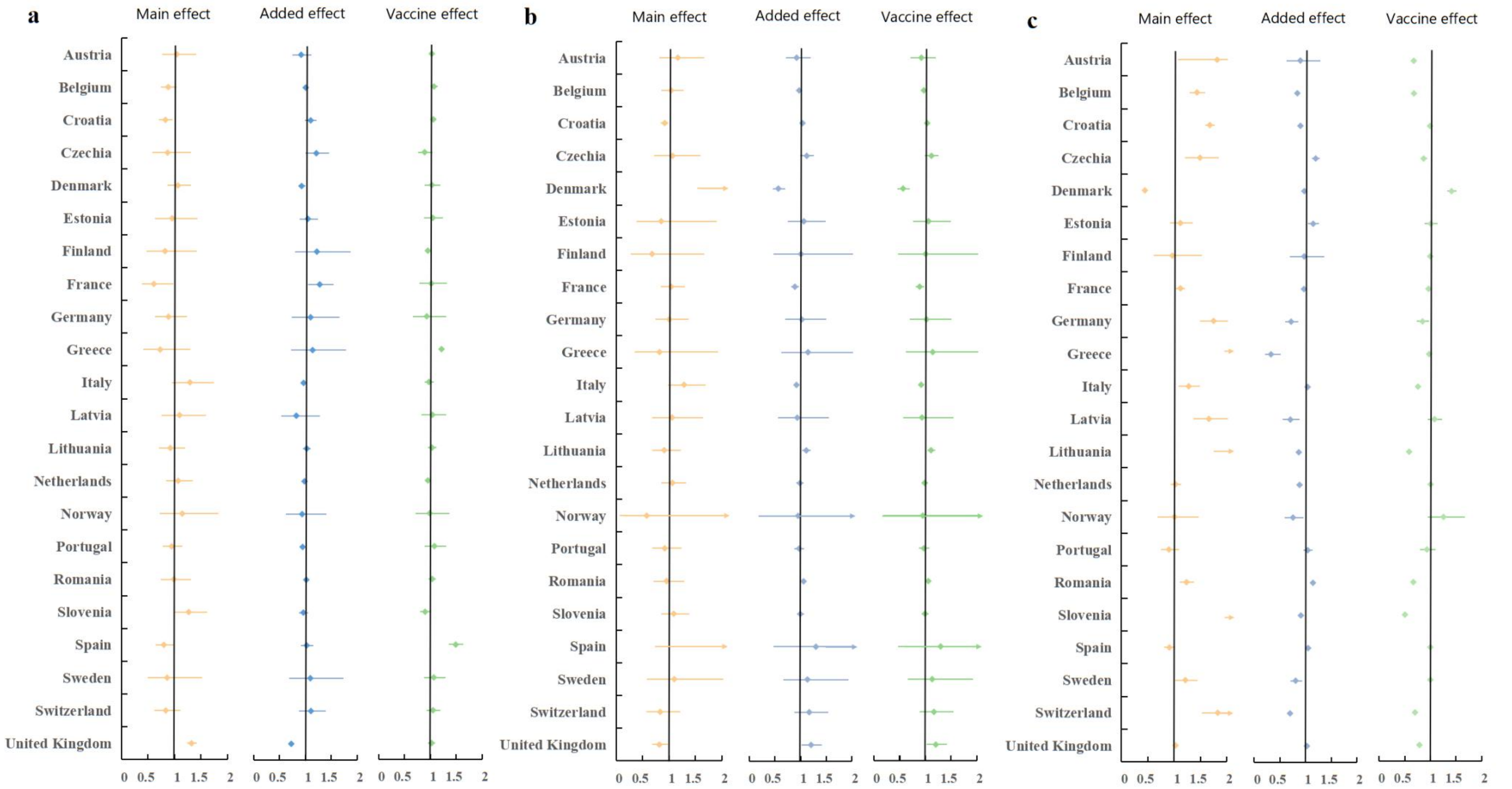

Figure S2. Main, added and vaccine effects for three outcomes in 22 countries under the weak intervention (above 60th percentile and lasts at least 7 days) after the vaccination rollouts during pandemic. (a) case growth rate, (b) death growth rate, (c) excess mortality.
